# Supplementary material for: Heterochronic activation of TGF-β signaling drives the diversity of the avian sterna
Source: Nat Commun. 2026 Apr 29;17:5877. doi: 10.1038/s41467-026-72602-6 (PMC13333996; doi:10.1038/s41467-026-72602-6)
Supplement: Supplementary file 1 — Supplementary Information [file 41467_2026_72602_MOESM1_ESM.pdf]

Supplementary Information

Heterochronic activation of TGF-β signaling drives the diversity of the avian sterna

Seung June Kwon et al.

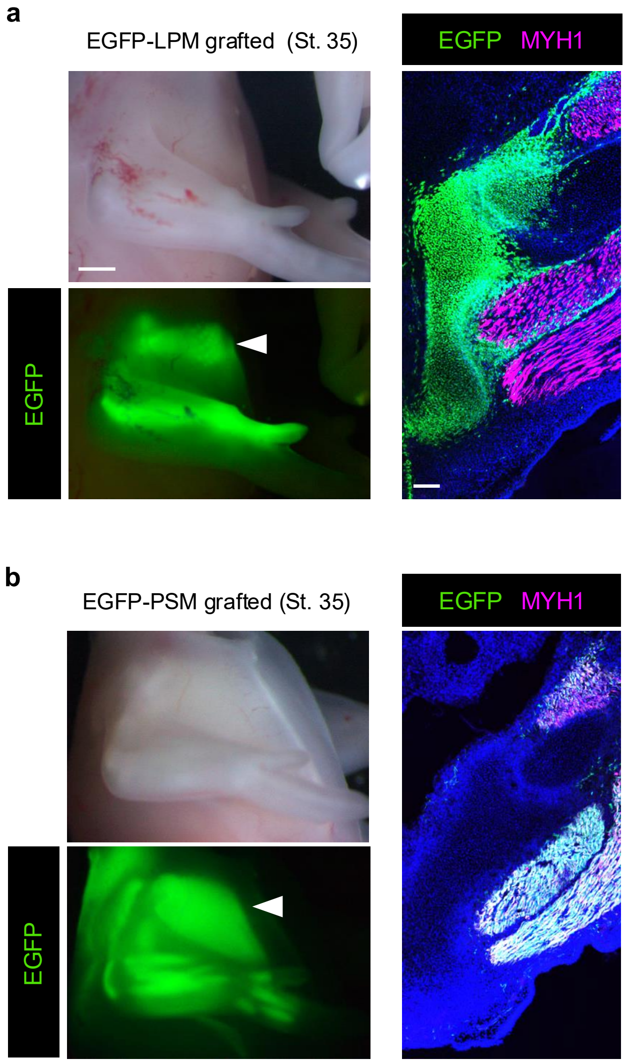

Supplementary Figure 1

Grafted EGFP-lateral plate mesoderm (LPM) contributed to the sternal region, whereas grafted EGFP-presomitic mesoderm (PSM) gave rise to the pectoral muscles

a A whole-mount view and transverse section of a St. 35 embryo grafted with EGFP-labeled LPM (see also Fig. 1e). Keel-forming EGFP-positive cells are indicated by an arrowhead. EGFP signals do not merge with MYH1 (magenta). **b** A whole-mount view and transverse section of a St. 35 embryo grafted with EGFP-labeled PSM. The grafted EGFP-positive cells differentiated into MYH1-positive pectoral muscle cells. Each experiment was repeated 3 times independently with similar results. Scale bars, 1 mm, 100 µm.

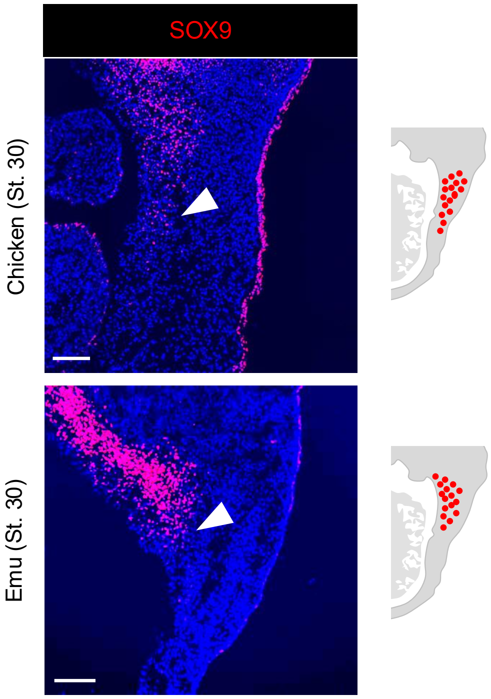

Supplementary Figure 2

SOX9 signals in the sternum-forming region in St. 30 chicken and emu

Signals for SOX9 proteins (red) were detected at the sternum forming region of St. 30 chicken and emu embryos (arrowheads). Each experiment was repeated 3 times independently with similar results. Scale bars, 200 µm.

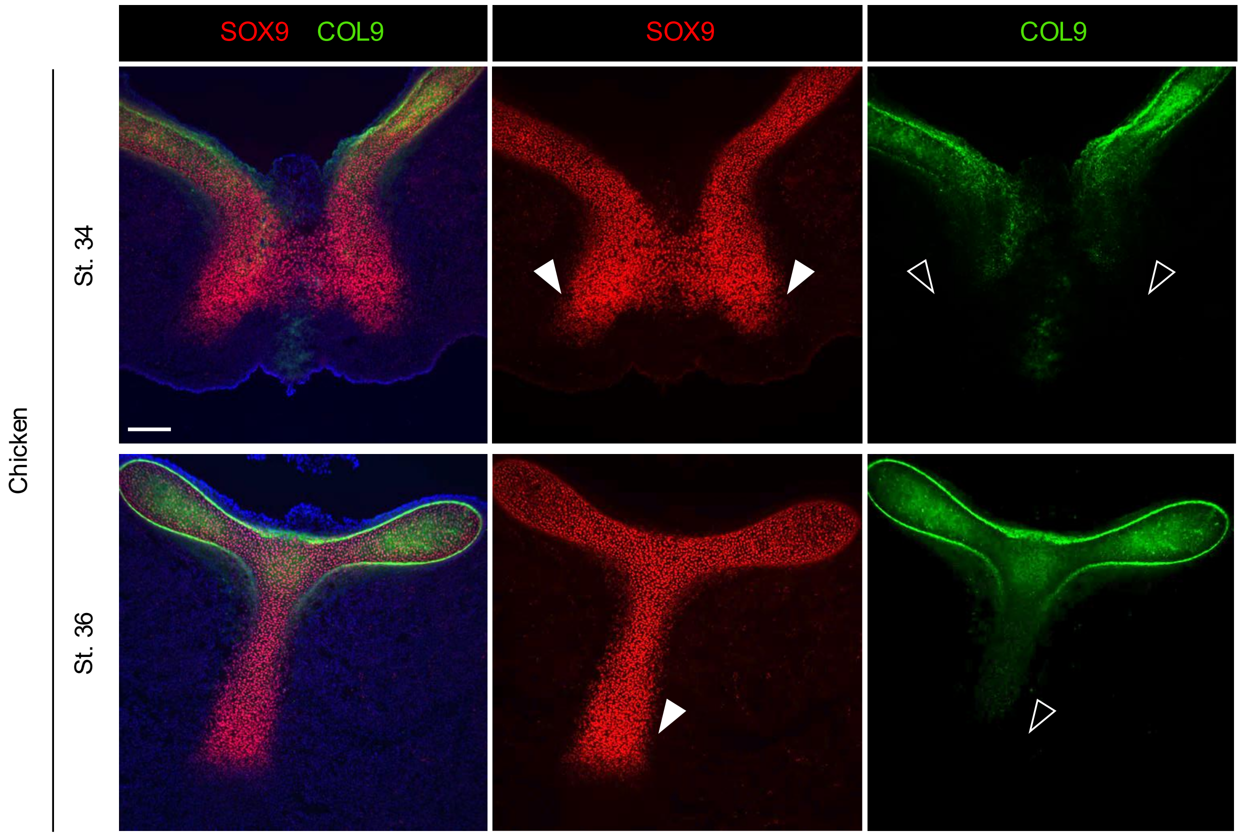

Supplementary Figure 3

Dorsal SPs have differentiated into mature, COL9^{+} chondrocytes in St. 34 and 36 chickens

Sternum cartilage of St. 34 and 36 chickens immunostained with antibodies against SOX9 (red) and collagen IX (COL9, green). At both stages, ventral SPs were SOX9-positive and COL9-negative (arrowheads and open arrowheads). Each experiment was repeated 3 times independently with similar results. Scale bar, 150 µm.

Sample	Total	Assigned	UMIs	Genes	UMIs/Genes
Chicken_st_24_1	3,218,625	214,752	16,382	6,494	2.52263628
Chicken_st_24_2	2,665,229	262,449	23,355	6,733	3.46873608
Chicken_st_24_3	2,072,181	366,874	38,772	7,146	5.4256927
Chicken_st_24_4	4,985,091	1,031,024	109,587	9,592	11.4248332
Chicken_st_36_d_1	3,556,246	893,431	133,781	9,266	14.4378373
Chicken_st_36_d_2	4,347,536	1,106,472	437,122	13,224	33.0552027
Chicken_st_36_d_3	888,111	169,813	89,758	9,426	9.52238489
Chicken_st_36_d_4	2,245,897	458,390	126,607	9,766	12.964059
Chicken_st_36_v_1	1,961,618	603,817	176,128	11,181	15.7524372
Chicken_st_36_v_2	7,609,300	2,257,978	221,049	12,165	18.1709001
Chicken_st_36_v_3	1,715,588	399,141	35,540	7,601	4.67570057
Chicken_st_36_v_4	2,550,106	585,898	92,126	10,411	8.84890981

sample	total	Assigned	UMIs	Genes	UMIs/Genes
Emu_st_24_1	5,930,167	1,424,349	399,533	12,737	31.3679
Emu_st_24_2	9,746,575	2,261,320	612,896	13,326	45.9925
Emu_st_24_3	3,761,034	904,796	269,185	11,654	23.0981
Emu_st_36_d_1	11,072,984	2,273,325	240,219	12,703	18.9104
Emu_st_36_d_2	1,230,909	186,255	22,093	6,209	3.55822
Emu_st_36_d_3	6,161,581	1,119,069	122,273	11,220	10.8978
Emu_st_36_v_1	2,071,622	385,308	29,588	6,771	4.36981
Emu_st_36_v_2	6,140,765	1,313,329	95,365	9,683	9.8487
Emu_st_36_v_3	3,113,776	592,419	41,439	7,492	5.5311

Supplementary Figure 4

The sequencing metrics of PIC-RNA-Seq

Numbers of total reads, assigned reads, unique molecular identifiers (UMIs), and detected genes are shown for each PIC-RNA-Seq samples.

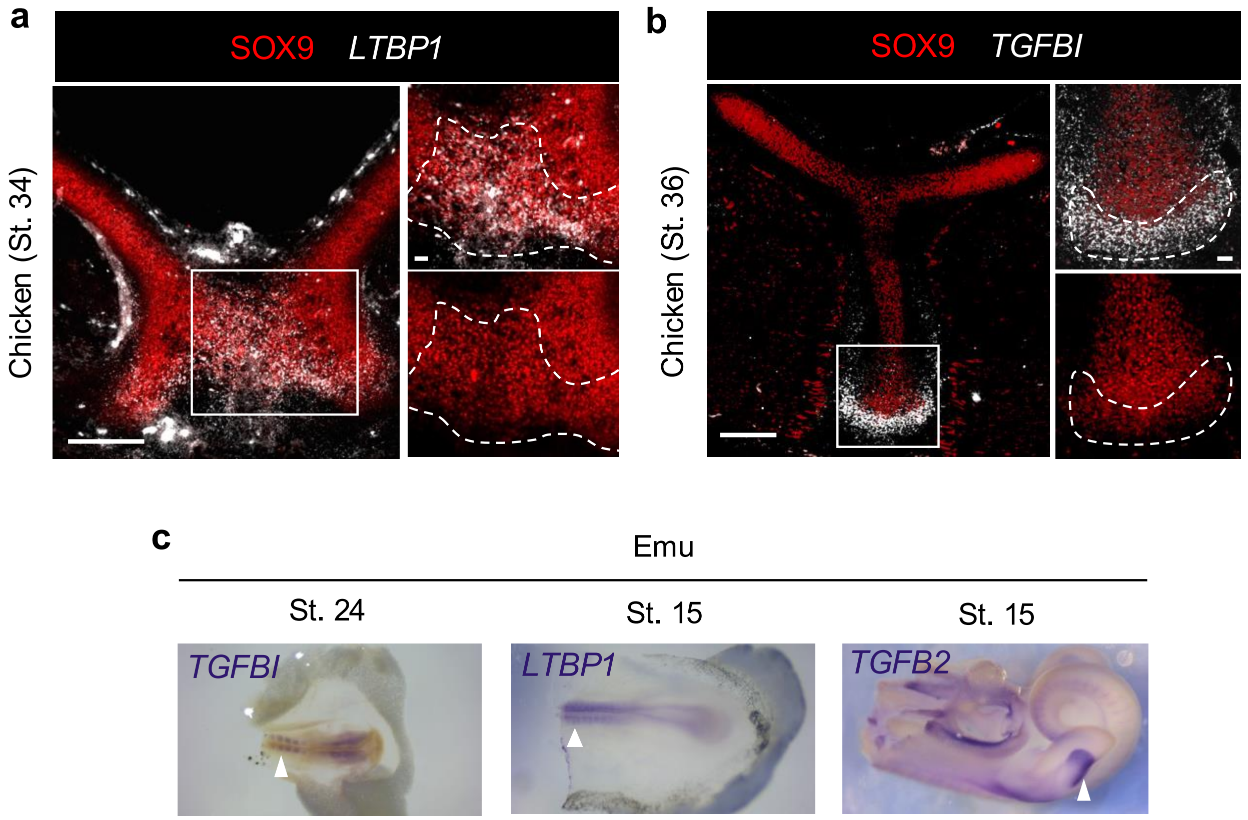

Supplementary Figure 5

Colocalization of SOX9 with *LTBP1* and *TGFBI* in chicken ventral SPs, and validation of emu riboprobes

Detection of SOX9 (red) with *LTBP1* and *TGFBI* mRNA (white) in St. 34 (**a**) and 36 (**b**) chickens. In the dotted regions of the ventral parts of the sterna, the two signals are co-localized. **c** Anti-sense riboprobes synthesized for emu *TGFBI*, *LTBP1*, and *TGF-β2* were validated by showing signals in somites at St. 15 (*TGFBI*, *LTBP1*) and in the limb mesenchyme at St. 24 (*TGF-β2*) (arrowheads), indicating that the probes worked normally. Experiments were repeated independently 3 times for **a** and **b**, and 2 times for **c**, with similar results. Scale bars, 200 μm; inset 30 μm.

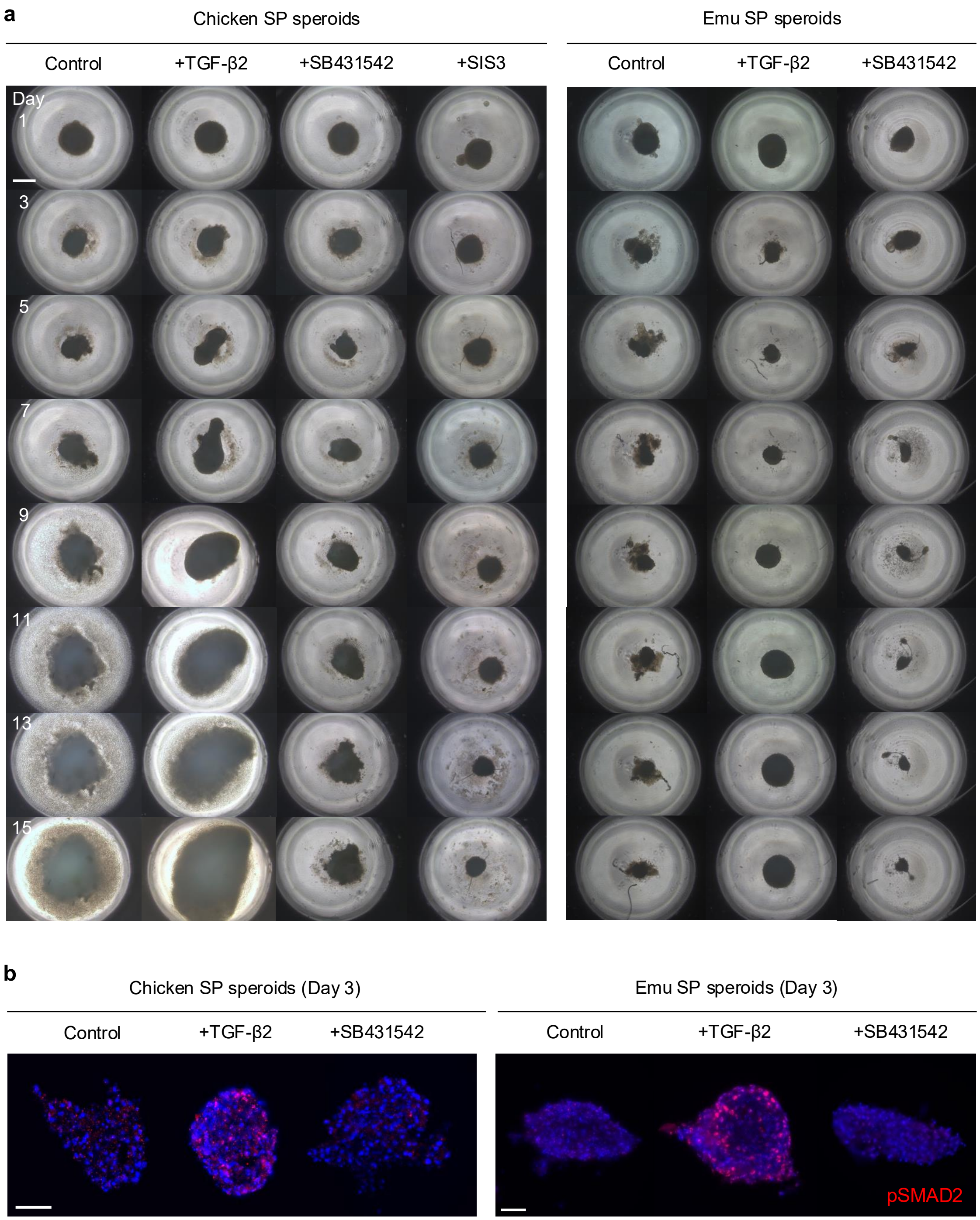

Supplementary Figure 6

Chicken and emu SP spheroid cultures

a Time course of spheroid morphological dynamics under each condition. **b** Detection of pSMAD2 (red) in day 3 chicken and emu SP spheroids. Experiments were repeated independently 5 times for **a**, and 3 times for **b**, with similar results. Scale bars, 500 μm (**a**), 50 μm (**b**).

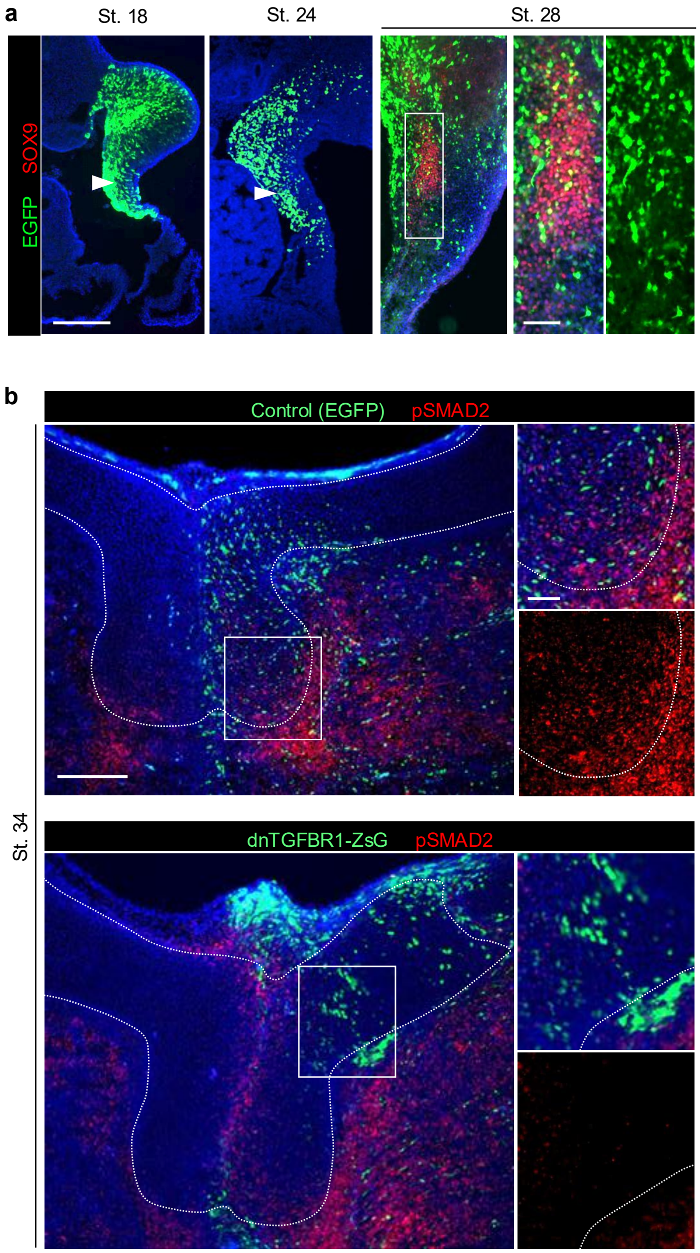

Supplementary Figure 7

Time course and pSMAD2 detection of electroporated SPs

a Time course of electroporated EGFP-expressing SPs, highlighted by arrowheads and the box. Immunostaining for SOX9 (red) was conducted at St. 28. **b** Detection of pSMAD2 (red) in St. 34 dnTGFBR1- and EGFP-expressing cells. pSMAD2 signal is abundant in control SPs but absent in dnTGFBR1-expressing SPs. Experiments were repeated 3 times independently with similar results. Scale bars, 200 µm; inset 50 µm.

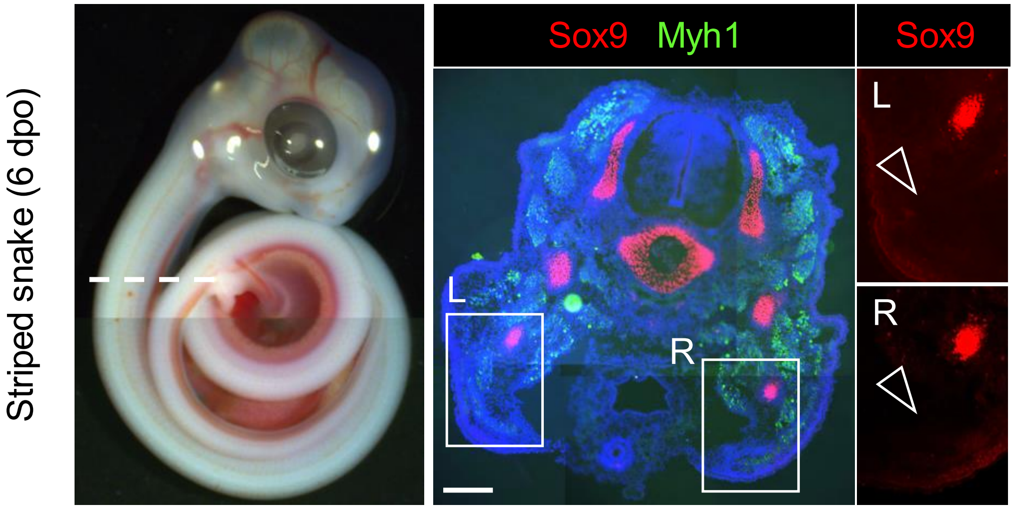

Supplementary Figure 8

Absence of SOX9-positive SPs in the snake embryo

A transverse section of the anterior thoracic region (7-8th somite level) of a Japanese striped snake embryo (n = 1) at 6 days post oviposition (6 DPO, developmentally comparable to chicken/emu St. 30), immunostained with antibodies against Sox9 (red) and Myh1 (cyan). Sox9 signals were absent in the ventral LPM regions on both sides (open arrowheads). Scale bar, 200 µm.
