# Supplementary material for: Heterochronic activation of TGF-β signaling drives the diversity of the avian sterna
Source: Nat Commun. 2026 Apr 29;17:5877. doi: 10.1038/s41467-026-72602-6 (PMC13333996; doi:10.1038/s41467-026-72602-6)
Supplement: Supplementary file 2 — Description of Additional Supplementary Files [file 41467_2026_72602_MOESM2_ESM.pdf]

Description of Additional Supplementary Files

File name: Supplementary Data 1
Description: PIC-RNA-Seq results in chicken and emu St. 36. PIC-RNA-Seq results in St. 36 chicken and emu SPs obtained by DESeq2, including gene ID, gene symbols, base mean, log2foldChange, stat and adjusted *p*-values.

File name: Supplementary Data 2
Description: Primer and probe sequences used for ISH and expression plasmid. Primer and probe sequences used for *TGFBI*, *LTBP1*, *TGFB2*, and dnTGFBR1.

File name: Supplementary Data 3
Description: PIC-RNA-Seq raw counts in chicken and emu.
Raw read counts from PIC-RNA-Seq of chicken and emu SPs.

File name: Supplementary Data 4
Description: Enriched GO terms at St. 36 chicken ventral SPs.
Enriched GO terms of St. 36_V chicken SPs analyzed by PANTHER overrepresentation test.

File name: Supplementary Movie 1
Description: Three-dimensional rendering of an adult chicken sternum in an orthographic view. 3-D movie of an adult chicken sternum generated using Amira by volume/surface rendering in a pseudo-color.

File name: Supplementary Movie 2
Description: Three-dimensional rendering of an adult emu sternum in an orthographic view. 3-D movie of an adult emu sternum generated using Amira by volume/surface rendering in a pseudo-color.
