# Supplementary material for: Heterochronic activation of TGF-β signaling drives the diversity of the avian sterna
Source: Nat Commun. 2026 Apr 29;17:5877. doi: 10.1038/s41467-026-72602-6 (PMC13333996; doi:10.1038/s41467-026-72602-6)
Supplement: Supplementary file 9 — Reporting Summary [file 41467_2026_72602_MOESM9_ESM.pdf]

nature portfolio

Corresponding author(s): Yuji Atsuta

Last updated by author(s): Apr 1, 2026

Reporting Summary

Nature Portfolio wishes to improve the reproducibility of the work that we publish. This form provides structure for consistency and transparency in reporting. For further information on Nature Portfolio policies, see our Editorial Policies and the Editorial Policy Checklist.

Statistics

For all statistical analyses, confirm that the following items are present in the figure legend, table legend, main text, or Methods section.

n/a	Confirmed	
☐	☒	The exact sample size (*n*) for each experimental group/condition, given as a discrete number and unit of measurement
☐	☒	A statement on whether measurements were taken from distinct samples or whether the same sample was measured repeatedly
☐	☒	The statistical test(s) used AND whether they are one- or two-sided<br>*Only common tests should be described solely by name; describe more complex techniques in the Methods section.*
☒	☐	A description of all covariates tested
☐	☒	A description of any assumptions or corrections, such as tests of normality and adjustment for multiple comparisons
☐	☒	A full description of the statistical parameters including central tendency (e.g. means) or other basic estimates (e.g. regression coefficient) AND variation (e.g. standard deviation) or associated estimates of uncertainty (e.g. confidence intervals)
☐	☒	For null hypothesis testing, the test statistic (e.g. *F*, *t*, *r*) with confidence intervals, effect sizes, degrees of freedom and *P* value noted<br>*Give P values as exact values whenever suitable.*
☒	☐	For Bayesian analysis, information on the choice of priors and Markov chain Monte Carlo settings
☒	☐	For hierarchical and complex designs, identification of the appropriate level for tests and full reporting of outcomes
☒	☐	Estimates of effect sizes (e.g. Cohen's *d*, Pearson's *r*), indicating how they were calculated

Our web collection on statistics for biologists contains articles on many of the points above.

Software and code

Policy information about availability of computer code

Data collection	RNA-seq libraries were prepared according to standard protocols and sequenced on an Illumina NovaSeq 6000 platform with paired-end 150 bp reads (PE150). UMIs and sample barcodes were extracted from the raw reads using UMI-tools (v1.0.0). The processed reads were aligned to the reference genomes obtained from Ensembl (chicken: GRCg6a, GCA_000002315.5; emu: ZJU2.0, GCA_016128335.2) using HISAT2 (v2.1.0).
Data analysis	For RNA-seq data analysis, read counts were quantified using featureCounts (v2.0.3). Differential gene expression analysis and principal component analysis (PCA) were performed using DESeq2 (v1.38.3), and volcano plots were generated accordingly. Box plots were created using normalized counts obtained from DESeq2 and visualized with ggplot2 (v3.4.0). Gene Ontology (GO) analysis was conducted using PANTHER (v18.0), and the top GO terms from the PANTHER hierarchy were represented in the plots. Protein–protein interaction networks were analyzed using STRING (v12.0) based on differentially expressed genes (DEGs) with an adjusted p-value $< 5 \times 10^{-6}$. Code availability:this study used previously published computational tools, with specific parameters detailed under https://github.com/seungjunekwon/avianSternum.

For manuscripts utilizing custom algorithms or software that are central to the research but not yet described in published literature, software must be made available to editors and reviewers. We strongly encourage code deposition in a community repository (e.g. GitHub). See the Nature Portfolio guidelines for submitting code & software for further information.

Data

Policy information about availability of data

All manuscripts must include a data availability statement. This statement should provide the following information, where applicable:

- Accession codes, unique identifiers, or web links for publicly available datasets
- A description of any restrictions on data availability
- For clinical datasets or third party data, please ensure that the statement adheres to our policy

Quantitative data from this study have been deposited to figshare (https://doi.org/10.6084/m9.figshare.30254737). PIC-RNA-Seq data is available in the GEO repository under accession number GSE313054.

Research involving human participants, their data, or biological material

Policy information about studies with human participants or human data. See also policy information about sex, gender (identity/presentation), and sexual orientation and race, ethnicity and racism.

Reporting on sex and gender	*Use the terms sex (biological attribute) and gender (shaped by social and cultural circumstances) carefully in order to avoid confusing both terms. Indicate if findings apply to only one sex or gender; describe whether sex and gender were considered in study design; whether sex and/or gender was determined based on self-reporting or assigned and methods used. Provide in the source data disaggregated sex and gender data, where this information has been collected, and if consent has been obtained for sharing of individual-level data; provide overall numbers in this Reporting Summary. Please state if this information has not been collected. Report sex- and gender-based analyses where performed, justify reasons for lack of sex- and gender-based analysis.*
Reporting on race, ethnicity, or other socially relevant groupings	*Please specify the socially constructed or socially relevant categorization variable(s) used in your manuscript and explain why they were used. Please note that such variables should not be used as proxies for other socially constructed/relevant variables (for example, race or ethnicity should not be used as a proxy for socioeconomic status). Provide clear definitions of the relevant terms used, how they were provided (by the participants/respondents, the researchers, or third parties), and the method(s) used to classify people into the different categories (e.g. self-report, census or administrative data, social media data, etc.) Please provide details about how you controlled for confounding variables in your analyses.*
Population characteristics	*Describe the covariate-relevant population characteristics of the human research participants (e.g. age, genotypic information, past and current diagnosis and treatment categories). If you filled out the behavioural & social sciences study design questions and have nothing to add here, write "See above."*
Recruitment	*Describe how participants were recruited. Outline any potential self-selection bias or other biases that may be present and how these are likely to impact results.*
Ethics oversight	*Identify the organization(s) that approved the study protocol.*

Note that full information on the approval of the study protocol must also be provided in the manuscript.

Field-specific reporting

Please select the one below that is the best fit for your research. If you are not sure, read the appropriate sections before making your selection.

☒ Life sciences ☐ Behavioural & social sciences ☐ Ecological, evolutionary & environmental sciences

For a reference copy of the document with all sections, see nature.com/documents/nr-reporting-summary-flat.pdf

Life sciences study design

All studies must disclose on these points even when the disclosure is negative.

Sample size	Sample sizes were based on standard practices in the field and previous studies using similar experimental systems. For 3-D culture, bead implantation, and electroporation experiments, typically five or more independent biological replicates per group were included to ensure reproducibility. For RNA-seq experiments, at least three independent biological replicates per condition were analyzed. No statistical methods were used to predetermine sample size.
Data exclusions	One sample (St. 36_D_4) from the emu St. 36 dorsal group was excluded due to extremely low read counts that did not meet quality control criteria. The original sample size for this group was four, and three samples were included in the final analysis.
Replication	All experiments were independently repeated with similar results. Representative images are shown, and similar results were observed across independent biological replicates.
Randomization	No randomization was performed, as samples were assigned to specific experimental groups based on developmental stage and sample type.

Blinding	No blinding was performed, as sample identity was required for analysis based on tissue type and developmental stage.

Reporting for specific materials, systems and methods

We require information from authors about some types of materials, experimental systems and methods used in many studies. Here, indicate whether each material, system or method listed is relevant to your study. If you are not sure if a list item applies to your research, read the appropriate section before selecting a response.

Materials & experimental systems

n/a	Involved in the study
☐	☒ Antibodies
☒	☐ Eukaryotic cell lines
☒	☐ Palaeontology and archaeology
☐	☒ Animals and other organisms
☒	☐ Clinical data
☒	☐ Dual use research of concern
☒	☐ Plants

Methods

n/a	Involved in the study
☒	☐ ChIP-seq
☒	☐ Flow cytometry
☒	☐ MRI-based neuroimaging

Antibodies

Antibodies used	Primary antibodies used in this study are: rabbit anti-pSMAD2(1:300, Cell Signaling, 138D4), rabbit anti-Sox9 for early chondrocytes (1:1000; Merck Millipore, AB5535), mouse anti-Myh1E for the myosin heavy chain (1:1500; DSHB, MF20), rabbit anti-pHH3 for mitosis (1:1000; Merck Millipore, 06-570), mouse anti-Col2a1 (DSHB, II-II6B3) and Col9a1 (DSHB, D1-9) for chondrocytes (1:500).
Validation	For GFP and ZsGreen detection, unelectroporated control embryos were used as negative controls. For antibody staining, samples incubated with secondary antibodies only were used as negative controls.

Animals and other research organisms

Policy information about studies involving animals; ARRIVE guidelines recommended for reporting animal research, and Sex and Gender in Research

Laboratory animals	Fertilized chicken, emu, and Japanese striped snake eggs were obtained from local vendors in Japan and from the Snake Research Center, and incubated to the desired developmental stages in a humidified incubator.
Wild animals	No wild animals were used in this study.
Reporting on sex	Embryos were not sexed prior to tissue collection or RNA extraction.
Field-collected samples	No field-collected samples were used in this study.
Ethics oversight	In accordance with institutional guidelines at Kyushu University and national regulations in Japan, formal ethical approval was not required because all experiments were performed using non-mammalian embryos (avian and reptilian) prior to hatching.

Note that full information on the approval of the study protocol must also be provided in the manuscript.

Plants

Seed stocks	*Report on the source of all seed stocks or other plant material used. If applicable, state the seed stock centre and catalogue number. If plant specimens were collected from the field, describe the collection location, date and sampling procedures.*
Novel plant genotypes	*Describe the methods by which all novel plant genotypes were produced. This includes those generated by transgenic approaches, gene editing, chemical/radiation-based mutagenesis and hybridization. For transgenic lines, describe the transformation method, the number of independent lines analyzed and the generation upon which experiments were performed. For gene-edited lines, describe the editor used, the endogenous sequence targeted for editing, the targeting guide RNA sequence (if applicable) and how the editor was applied.*
Authentication	*Describe any authentication procedures for each seed stock used or novel genotype generated. Describe any experiments used to assess the effect of a mutation and, where applicable, how potential secondary effects (e.g. second site T-DNA insertions, mosiacism, off-target gene editing) were examined.*
