# Supplementary material for: Heterochronic activation of TGF-β signaling drives the diversity of the avian sterna
Source: Nat Commun. 2026 Apr 29;17:5877. doi: 10.1038/s41467-026-72602-6 (PMC13333996; doi:10.1038/s41467-026-72602-6)
Supplement: Supplementary file 10 — Transparent Peer Review file [file 41467_2026_72602_MOESM10_ESM.pdf]

nature portfolio

Peer Review File

Heterochronic activation of TGF-β signaling drives the diversity of the avian sterna

Corresponding Author: Dr Yuji Atsuta

This file contains all reviewer reports in order by version, followed by all author rebuttals in order by version.

Version 0:

Reviewer comments:

Reviewer #1

(Remarks to the Author)
Kwon et al. investigated the molecular mechanisms underlying the sternum diversity among avians. Although the diversity of keel morphology associated with avian flight ability is well known, the genes and signaling pathways that generate this diversity remain uninvestigated. The authors found that chondrocyte differentiation proceeds faster in emus than chickens, generating the shorter keel. Then using RNA-seq, they identified TGF-B signaling as one of the pathways responsible for keel diversity. Finally, functional tests using 3D spheroids and bead implantations were conducted to test TGF-B signaling. The overall scheme and logic are coherent and well established. The finding in this paper adds valuable empirical knowledge to evolutionary developmental biology. However, before further consideration, this paper needs some major and minor improvements. Some observations and descriptions of the ISH staining are not sufficient to lead to reliable conclusions and provide novel insights into skeletal evolution. Several sentences lack scientific clarity and accuracy. See the specific comments below.

Major.

Fig. 4
The ISH staining of each probe provides a conspicuous signal in the central region of the keel. However, the authors need to present photos with higher magnification and overlapping images of Sox9 and TGFBI, LTBP1, and TGFB2. The questions are where exactly these genes are expressed during chondrogenesis; proliferating or resting chondrocytes? Perichondrium? How are their expressions overlapped or separated? More detailed observations and descriptions of these major players in keel diversity should help better understand and dissect the underlying molecular mechanisms. A discussion of their precise localizations and possible functions in evolution is also critical. To this end, double staining of these gene expressions would be a preferable method. Alternatively, if there are any technical difficulties, the authors may stain these gene expressions individually using successive tissue sections, so that they can compare these genes' localizations.

Page 15 line 23
The authors mentioned that the left and right SP2 migrate towards the midline and fuse, developing the keel. However, the author did not provide any evidence whether there is actual cell migration or the expansion of cell differentiation towards the midline. These two possibilities are fundamentally different; therefore, they should be tested precisely. As the authors are finding the possibility of cell migration defects in Fig.6, they should conduct live imaging or at least cell fate tracking by expressing dominant-negative or constitutively active TGF-B receptor 1 and EGFP in the same cells. These additional experiments are required to support the current interpretations in the Discussion.

Minor.

P2, line 4
"The skeletal template of vertebrates is established during embryogenesis through intramembranous ossification in the head and endochondral ossification in the trunk."
This statement is not scientifically accurate. In vertebrates, the head has many endochondral bones and the trunk does intramembranous bones.

P2, line 9-10
There are many excellent papers that have demonstrated the molecular and genetic mechanisms underlying interspecies phenotypic differences. The authors should cite more papers from the broad field of skeletal evolutionary developmental biology and can provide a broader view of the recent progress.
For example:
Saxena A, Sharma V, Muthuirulan P, Neufeld SJ, Tran MP, Gutierrez HL, Chen KD, Erberich JM, Birmingham A, Capellini TD, Cobb J, Hiller M, Cooper KL. Interspecies transcriptomics identify genes that underlie disproportionate foot growth in jerboas. Curr Biol. 2022 Jan 24;32(2):289-303.e6.
Chan YF, Marks ME, Jones FC, Villarreal G Jr, Shapiro MD, Brady SD, Southwick AM, Absher DM, Grimwood J, Schmutz J, Myers RM, Petrov D, Jónsson B, Schluter D, Bell MA, Kingsley DM. Adaptive evolution of pelvic reduction in sticklebacks by recurrent deletion of a Pitx1 enhancer. Science. 2010 Jan 15;327(5963):302-5.

P4, Line10
Prrx-cre is expressed in the entire limb bud at the early stage. This transgenic is not “SP-specific Cre”. The statement should be modified and scientifically accurate.

P4, line 12-16
“However, Runx1/2 and Sox9 are master regulators of osteogenesis and chondrogenesis, respectively, and are essential for the formation of nearly all bones and cartilage.”

I totally agree with this statement itself. However, phenotypic changes may occur by gene expression changes (including heterochrony as analyzed in this paper), modifying pre-existing cis regulatory activities or generating de novo cis regulatory regions. Expression of these “master” regulators may be modified only in the prospective keel domain without affecting other regions, and, consequently, the keel morphology may change. Thus, I would not agree with the logic presented in this paragraph supposing “master and essential regulators do not change for a modification of one bone”.

Page 8, line 5
“mRNA information”. I believe that the authors intended to refer to “transcriptomic profiles” or something similar.

Page 9, line 11
“TGFBI, Fibulin1(FBLN1), Collagen1A2
(COL1A2), GJA1 (also known as connexin43), SOX9, and PITX2 were particularly high….”
This should be “The expression levels of TGFBI, Fibulin1(FBLN1), Collagen1A2
(COL1A2), GJA1 (also known as connexin43), SOX9, and PITX2 were particularly high ……” or something equivalent.

Page 9, line 18
“positively regulates the organization”
This expression is unclear. What is the meaning of “positively” and “organization” in this context?

Page 19, line 2- “gallus” should be italic.

(Remarks on code availability)

Reviewer #2

(Remarks to the Author)
The manuscript “Heterochronic activation of TGF-B signaling drives the diversity of the avian sterna” Kwon et al. describe a set of experiments that compare the development of the sternum between flight capable chickens and flightless emus. Flight capable birds form a ventral protrusion from the sternum referred to as the keel allowing for flight muscle attachment. In contrast, flightless birds form a flat sternum. The authors first show that both the chicken and the emu initiate sternum formation at equivalent stages and that Sox9 positive cells from the LPM fuse ventrally also at equivalent stages. However, the chick sternum cells continue to proliferate producing the ventral keel whereas the emu sternum forms mature chondrocytes. Using comparative transcriptomics, the authors find TGF-B is uniquely expressed in the chick keel. Using an in vitro assay, they find TGF-B signaling is both necessary and sufficient for sternal growth in both species. Finally, using small molecule and dominant negative approaches, the authors show TGF-B is necessary for keel formation in the chick in vivo.
This study is of great interest to developmental biologist and evolutionary biologist as it provides novel insight into sternum formation and sheds light on how flight loss in birds results in morphological changes to the skeleton. The experiments are

well-controlled and thorough. Only minor revisions are needed before I recommend publication.
1. The authors provide a compelling case that TGF-B signaling is required for keel formation. This is shown by the presence of TGF-B transcripts and phenotypic effects of small molecule inhibition or over-expressing a dominant negative TGFBR1. I would recommend the authors confirm specific TGF-B inhibition by phospho-smad2/3 staining to demonstrate reduced TGF-B-signaling. This will have the further benefit of showing where active signaling is occurring in unmanipulated embryos.
2. On page 13 of the manuscript, the authors state that the SPs require cell-autonomous TGF-B signaling. Figure 4 shows expression of the TGF-B ligands and figure S5 shows co-localization of Sox9. However, the expression of TGF-B appears to extend beyond the SPs. It's difficult to determine if the SPs are not responding to their cellular environment. Figure 6f shows that cells without functioning receptor don't localize to the keel. Is it possible that these cells are unable to respond to extrinsic signals emanating from outside the SPs. I'd recommend softening the language or determining if the signal is cell autonomous through further experimentation. This could be done by transplanting the chick LPM into the emu and ask if the keel still forms. However, given the difficulty of procuring fertile emus, I'd recommend the former.
3. It's unclear if the pHH3 quantification was done using multiple sections from one embryo or collected from multiple individuals. Please clarify.
4. Supplementary figure S2 would benefit from a schematic showing where the image is from (like what the authors did in Figure 1d).

(Remarks on code availability)

Reviewer #3

(Remarks to the Author)
This is an exceptionally interesting and beautifully-executed study on the development of the keel on the breastbone (sternum) of birds that can or cannot fly (the latter being the flightless ratites). The sternal keel is one of the attachment points for the pectoral muscles that play a major part in driving the down-stroke of the wing during flight. It is particularly strongly-developed in birds that are dependent on exceptional flying performance, such as hummingbirds; and poorly-developed or absent in flightless birds. This study used the emu, a species vary rarely studied, and the chicken, a standard model species that has the ability to fly.

Using expression profiling, in vitro analyses of sternal progenitor growth, and microbead and tissue implants, the authors conclude that the TGF-beta singalling pathway shuts of in the emu before the sternal progenitors can build a keel, where in the chick, TGF beta signalling is extended so that the keel is built. This is an example of heterochrony, or a change in developmental timing. more specifically a type of heterochrony called peramorphosis.

This is a very high quality evo-devo study with excellent data, analysis and figures. It shows how changes in developmental timing can produce changes in the morphological phenotype. One doesn't often see such high quality evo devo studies like this, in which the authors' hypothesis is supported by multiple lines of cutting-edge experimental evidence, and so our confidence in the hypothesis is high.

This study is practically flawless so I struggle to find any points for improvement.

Minor points.

I couldn't find a citation of the Tabin study of Darwin's finches which showed that TGF beta signalling is involved in beak evolution. The parallels with present study are strong, so the authors might consider citing it if they want to.

The authors don't explain that what they have found is peramorphosis - a condition where growth is continued to a later stage than occurred in the ancestor.

The authors don't cite any reviews on heterochrony. They mention two experimental studies on the topic but many readers will not know much about heterochrony. So at least cite some reviews. E.g. the classic Gould 1977 Ontogeny and Phylogeny; or Syst Biol. 2005 Apr;54(2):230-40. doi: 10.1080/10635150590923227; or Semin Cell Dev Biol. 2014 Oct;34:99-107. doi: 10.1016/j.semcdb.2014.06.015.

(Remarks on code availability)

Version 1:

Reviewer comments:

Reviewer #1

(Remarks to the Author)
The authors have fully resolved my concerns and substantially improved the manuscript. The latest data well support the presented story and hypotheses. I appreciate the effort made by the authors during the revise period. This paper should be promptly published in Nature Communications.

(Remarks on code availability)

Reviewer #2

(Remarks to the Author)
My concerns have been addressed and I recommend publication.

(Remarks on code availability)

Reviewer #3

(Remarks to the Author)
The authors have made a more than adequate rebuttal to the reviewers and so I now recommend publication without further revision.

(Remarks on code availability)
I haven't reviewed the code because this is outside my area of expertise.

Open Access This Peer Review File is licensed under a Creative Commons Attribution 4.0 International License, which permits use, sharing, adaptation, distribution and reproduction in any medium or format, as long as you give appropriate credit to the original author(s) and the source, provide a link to the Creative Commons license, and indicate if changes were made.
In cases where reviewers are anonymous, credit should be given to 'Anonymous Referee' and the source.
The images or other third party material in this Peer Review File are included in the article's Creative Commons license, unless indicated otherwise in a credit line to the material. If material is not included in the article's Creative Commons license and your intended use is not permitted by statutory regulation or exceeds the permitted use, you will need to obtain permission directly from the copyright holder.
To view a copy of this license, visit https://creativecommons.org/licenses/by/4.0/

Point-by-Point Response to the Reviewers

Reviewer #1

Kwon et al. investigated the molecular mechanisms underlying the sternum diversity among avians. Although the diversity of keel morphology associated with avian flight ability is well known, the genes and signaling pathways that generate this diversity remain uninvestigated. The authors found that chondrocyte differentiation proceeds faster in emus than chickens, generating the shorter keel. Then using RNA-seq, they identified TGF-B signaling as one of the pathways responsible for keel diversity. Finally, functional tests using 3D spheroids and bead implantations were conducted to test TGF-B signaling. The overall scheme and logic are coherent and well established. The finding in this paper adds valuable empirical knowledge to evolutionary developmental biology. However, before further consideration, this paper needs some major and minor improvements. Some observations and descriptions of the ISH staining are not sufficient to lead to reliable conclusions and provide novel insights into skeletal evolution. Several sentences lack scientific clarity and accuracy. See the specific comments below.

We thank the reviewer for their thoughtful evaluation of our manuscript and for recognizing the overall coherence of the experimental design and the significance of our findings. We are particularly encouraged that the reviewer considers our study to provide valuable insights into the developmental mechanisms underlying skeletal diversification. Indeed, one of the central aims of this work was to elucidate how temporal modulation of signaling pathways contributes to morphological diversification of homologous skeletal structures, and we agree that our findings provide important empirical insight into this fundamental question in Evo-Devo biology.

Major.

Fig. 4
The ISH staining of each probe provides a conspicuous signal in the central region of the keel. However, the authors need to present photos with higher magnification and overlapping images of Sox9 and TGFBI, LTBP1, and TGFB2. The questions are where exactly these genes are expressed during chondrogenesis; proliferating or resting chondrocytes? Perichondrium? How are their expressions overlapped or separated? More detailed observations and descriptions of these major players in keel diversity should help better understand and dissect the underlying molecular mechanisms. A discussion of their precise localizations and possible functions in evolution is also critical. To this end, double staining of these gene expressions would be a preferable method. Alternatively, if there are any technical difficulties, the authors

may stain these gene expressions individually using successive tissue sections, so that they can compare these genes' localizations.

We thank the reviewer for this constructive suggestion. To precisely determine the localization of TGF-β pathway components, we performed multiplexed HCR combined with immunohistochemistry. These analyses revealed clear co-localization of SOX9 with *TGFBI* and *TGF-β2* within ventral SPs in both chicken and emu (Fig. 4b, d). In addition, *LTBP1* expression was detected in the same ventral SP domain, consistent with our transcriptomic results. These data clarify that activation of TGF-β signaling occurs within immature ventral SPs rather than being restricted to surrounding perichondrial regions.

We have included higher-magnification images and expanded the Results and Discussion sections to describe these localization patterns and their relevance to keel development and the heterochronic differences observed between species.

Page 15 line 23
The authors mentioned that the left and right SP2 migrate towards the midline and fuse, developing the keel. However, the author did not provide any evidence whether there is actual cell migration or the expansion of cell differentiation towards the midline. These two possibilities are fundamentally different; therefore, they should be tested precisely. As the authors are finding the possibility of cell migration defects in Fig.6, they should conduct live imaging or at least cell fate tracking by expressing dominant-negative or constitutively active TGF-B receptor 1 and EGFP in the same cells. These additional experiments are required to support the current interpretations in the Discussion.

We thank the reviewer for emphasizing the distinction between active migration and expansion. To address this point, we performed time-course fate mapping of EGFP-electroporated SPs. EGFP was introduced into SPs at St. 14, and their positions were examined at St. 18, 24, 28, 34, and 36 (Supplementary Fig. 7a; Fig. 6e). We found that labeled SPs progressively relocated toward the ventral midline and were incorporated into the elongating keel. In contrast, dnTGFBR1-expressing cells failed to integrate into the keel region not only at St. 36 but already at St. 34, remaining preferentially in dorsal regions. These data suggest that TGF-β signaling influences the ventral positioning of SPs during keel formation.

While we did not perform live imaging of individual cell trajectories, this temporal fate mapping analysis suggests that TGF-β signaling regulates SP positioning in addition to proliferation. We have revised the Discussion (page 16, line 20–page 17, line 9) to clarify this interpretation.

Constitutive activation of TGF-β signaling resulted in chondrogenic hyperplasia and embryonic lethality due to hernia (Fig. R1), precluding detailed positional analysis but supporting a critical requirement for tightly regulated TGF-β activity during sternal development.

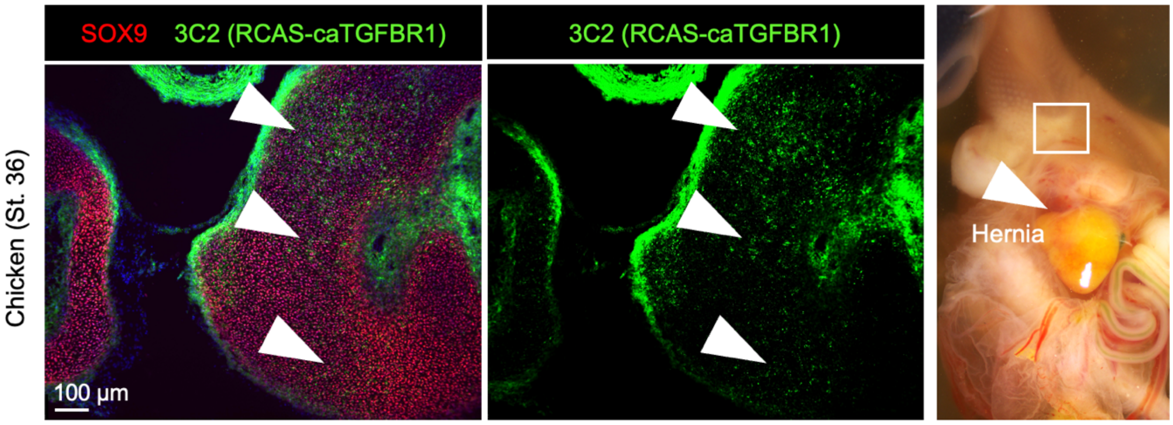

Fig. R1: Constitutive activation of TGF-β leads to chondrogenic hyperplasia and lethal hernia

Minor.

P2, line 4
"The skeletal template of vertebrates is established during embryogenesis through intramembranous ossification in the head and endochondral ossification in the trunk."
This statement is not scientifically accurate. In vertebrates, the head has many endochondral bones and the trunk does intramembranous bones.

We thank the reviewer for this correction. We have changed the statement to accurately reflect that both endochondral and intramembranous ossification contribute to both the cranial and postcranial skeleton.

P2, line 9-10
There are many excellent papers that have demonstrated the molecular and genetic mechanisms underlying interspecies phenotypic differences. The authors should cite more papers from the broad field of skeletal evolutionary developmental biology and can provide a broader view of the recent progress.
For example:
Saxena A, Sharma V, Muthuirulan P, Neufeld SJ, Tran MP, Gutierrez HL, Chen KD, Erberich JM, Birmingham A, Capellini TD, Cobb J, Hiller M, Cooper KL. Interspecies transcriptomics identify genes that underlie disproportionate foot growth in jerboas. Curr Biol. 2022 Jan 24;32(2):289-303.e6.
Chan YF, Marks ME, Jones FC, Villarreal G Jr, Shapiro MD, Brady SD, Southwick AM, Absher DM, Grimwood J, Schmutz J, Myers RM, Petrov D, Jónsson B, Schluter D, Bell MA,

Kingsley DM. Adaptive evolution of pelvic reduction in sticklebacks by recurrent deletion of a Pitx1 enhancer. Science. 2010 Jan 15;327(5963):302-5.

We thank the reviewer for this constructive suggestion. We have now incorporated the recommended studies (Saxena et al., 2022; Chan et al., 2010) and updated the Introduction to reflect recent progress in the field, providing a broader perspective on skeletal Evo-Devo.

P4, Line10
Prrx-cre is expressed in the entire limb bud at the early stage. This transgenic is not "SP-specific Cre". The statement should be modified and scientifically accurate.

We thank the reviewer for this important clarification. We have revised the sentence as "gene knockout (KO) can be achieved in LPM-derived mesenchyme, including SPs".

P4, line 12-16
"However, Runx1/2 and Sox9 are master regulators of osteogenesis and chondrogenesis, respectively, and are essential for the formation of nearly all bones and cartilage."
I totally agree with this statement itself. However, phenotypic changes may occur by gene expression changes (including heterochrony as analyzed in this paper), modifying pre-existing cis regulatory activities or generating de novo cis regulatory regions. Expression of these "master" regulators may be modified only in the prospective keel domain without affecting other regions, and, consequently, the keel morphology may change. Thus, I would not agree with the logic presented in this paragraph supposing "master and essential regulators do not change for a modification of one bone".

We agree with the reviewer that "master regulators" can indeed contribute to morphological diversification through changes in their spatiotemporal expression, such as via modifications of cis-regulatory elements.
We thank the reviewer for this important comment. We agree that modifications in the spatial or temporal regulation of master transcription factors could, in principle, contribute to morphological diversification without eliminating the entire structure. We have therefore revised the paragraph to clarify our logic. Specifically, we now state that *Runx1/2* and *Sox9* mutant mice exhibit complete loss of the sternum, indicating that these factors are essential for sternal formation; however, because the entire structure is absent in these mutants, it remains unclear whether these transcription factors regulate morphological diversification of the sternum and keel.

Page 8, line 5
"mRNA information". I believe that the authors intended to refer to "transcriptomic profiles"

or something similar.

We thank the reviewer for pointing this out and we have replaced the words to "transcriptomic profiles".

Page 9, line 11
"TGFBI, Fibulin1(FBLN1), Collagen1A2
(COL1A2), GJA1 (also known as connexin43), SOX9, and PITX2 were particularly high…."
This should be "The expression levels of TGFBI, Fibulin1(FBLN1), Collagen1A2
(COL1A2), GJA1 (also known as connexin43), SOX9, and PITX2 were particularly high
……" or something equivalent.

We thank the reviewer for this correction. We have revised the sentence as the reviewer's suggestion.

Page 9, line 18
"positively regulates the organization"
This expression is unclear. What is the meaning of "positively" and "organization" in this context?

We thank the reviewer for pointing this out. We have revised the text to clarify our meaning by replacing "positively regulates the organization" with "regulates the structural organization," which more precisely reflects the biological processes inferred from our GO and network analyses.

Page 19, line 2- "gallus" should be italic.

We thank the reviewer for pointing this out. This has been corrected.

Reviewer #2

The manuscript "Heterochronic activation of TGF-B signaling drives the diversity of the avian sterna" Kwon et al. describe a set of experiments that compare the development of the sternum between flight capable chickens and flightless emus. Flight capable birds form a ventral protrusion from the sternum referred to as the keel allowing for flight muscle attachment. In contrast, flightless birds form a flat sternum. The authors first show that both the chicken and the emu initiate sternum formation at equivalent stages and that Sox9 positive cells from the LPM fuse ventrally also at equivalent stages. However, the chick sternum cells

continue to proliferate producing the ventral keel whereas the emu sternum forms mature chondrocytes. Using comparative transcriptomics, the authors find TGF-B is uniquely expressed in the chick keel. Using an in vitro assay, they find TGF-B signaling is both necessary and sufficient for sternal growth in both species. Finally, using small molecule and dominant negative approaches, the authors show TGF-B is necessary for keel formation in the chick in vivo.

This study is of great interest to developmental biologist and evolutionary biologist as it provides novel insight into sternum formation and sheds light on how flight loss in birds results in morphological changes to the skeleton. The experiments are well-controlled and thorough. Only minor revisions are needed before I recommend publication.

We appreciate the reviewer's careful and encouraging assessment of our work. We are grateful for the positive evaluation of the experimental design and for recognizing that our comparative approach provides new insight into the developmental basis of sternum morphology and its evolutionary diversification. We have addressed the points raised and revised the manuscript accordingly.

1. The authors provide a compelling case that TGF-B signaling is required for keel formation. This is shown by the presence of TGF-B transcripts and phenotypic effects of small molecule inhibition or over-expressing a dominant negative TGFBR1. I would recommend the authors confirm specific TGF-B inhibition by phospho-smad2/3 staining to demonstrate reduced TGF-B-signaling. This will have the further benefit of showing where active signaling is occurring in unmanipulated embryos.

We thank the reviewer for this constructive suggestion. We have now included phospho-SMAD2 staining to confirm pathway activation and inhibition. In electroporation experiments, pSMAD2 levels were reduced in dnTGFBR1-expressing SPs, confirming specific inhibition of TGF-β signaling and revealing the spatial distribution of active signaling in SPs (Supplementary Fig. 7b).

In addition, in both chicken and emu SP spheroids, pSMAD2 levels increased upon TGF-β2 treatment and were reduced following SB-431542 treatment (Supplementary Fig. 6b). Corresponding to pSMAD2 levels, not only chicken spheroids but also emu spheroids showed significant reduction in spheroid size (Fig. 5c, d; Supplementary Fig. 6a).

2. On page 13 of the manuscript, the authors state that the SPs require cell-autonomous TGF-B signaling. Figure 4 shows expression of the TGF-B ligands and figure S5 shows co-localization of Sox9. However, the expression of TGF-B appears to extend beyond the SPs. It's difficult to determine if the SPs are not responding to their cellular environment. Figure 6f shows that cells without functioning receptor don't localize to the keel. Is it possible that

these cells are unable to respond to extrinsic signals emanating from outside the SPs. I'd recommend softening the language or determining if the signal is cell autonomous through further experimentation. This could be done by transplanting the chick LPM into the emu and ask if the keel still forms. However, given the difficulty of procuring fertile emus, I'd recommend the former.

We thank the reviewer for this thoughtful comment. We agree that our data do not exclude the possibility that SPs respond to extrinsic TGF-β signals emanating from surrounding cells. Although dnTGFBR1-expressing cells fail to localize to the keel region and show reduced levels of pSMAD2, these results demonstrate that TGF-β signaling within SPs is required for their contribution to the keel, but do not definitively distinguish between strictly cell-autonomous versus environmental effects. We have therefore softened the wording in the Results to avoid overinterpretation.

In addition, we performed multiplex HCR combined with immunohistochemistry to further investigate the source of the signal, and demonstrated co-localization of *TGF-β2*, *TGFBI*, and SOX9 within individual ventral SPs (Fig. 4b, 4d). While this suggests that autocrine signaling likely plays a role in TGF-β activation, we have updated the manuscript to acknowledge the potential contribution of the surrounding environment.

3. It's unclear if the pHH3 quantification was done using multiple sections from one embryo or collected from multiple individuals. Please clarify.

We thank the reviewer for pointing out this lack of clarity. The pHH3 quantification was performed using three sections from the central region of the sternum/keel or spheroid for each embryo or spheroid, and we have now clarified this point in the Methods section.

4. Supplementary figure S2 would benefit from a schematic showing where the image is from (like what the authors did in Figure 1d).

We thank the reviewer for this suggestion, and a schematic has now been added to Fig. S2.

<u>Reviewer #3</u>

This is an exceptionally interesting and beautifully-executed study on the development of the keel on the breastbone (sternum) of birds that can or cannot fly (the latter being the flightless ratites). The sternal keel is one of the attachment points for the pectoral muscles that play a

major part in driving the down-stroke of the wing during flight. It is particularly strongly-developed in birds that are dependent on exceptional flying performance, such as hummingbirds; and poorly-developed or absent in flightless birds. This study used the emu, a species vary rarely studied, and the chicken, a standard model species that has the ability to fly.

Using expression profiling, in vitro analyses of sternal progenitor growth, and microbead and tissue implants, the authors conclude that the TGF-beta singalling pathway shuts of in the emu before the sternal progenitors can build a keel, where in the chick, TGF beta signalling is extended so that the keel is built. This is an example of heterochrony, or a change in developmental timing. more specifically a type of heterochrony called peramorphosis.

This is a very high quality evo-devo study with excellent data, analysis and figures. It shows how changes in developmental timing can produce changes in the morphological phenotype. One doesn't often see such high quality evo devo studies like this, in which the authors' hypothesis is supported by multiple lines of cutting-edge experimental evidence, and so our confidence in the hypothesis is high.

This study is practically flawless so I struggle to find any points for improvement.

We sincerely appreciate the reviewer’s exceptionally positive evaluation of our study. We are grateful for their recognition of the significance of our findings and for highlighting the strength of our experimental approaches and analyses. We are pleased that the reviewer considers our work to provide a clear example of how heterochronic changes in developmental signaling contribute to morphological diversification.

Minor points.

I couldn't find a citation of the Tabin study of Darwin's finches which showed that TGF beta signalling is involved in beak evolution. The parallels with present study are strong, so the authors might consider citing it if they want to.

We thank the reviewer for this helpful suggestion and we have now cited Abzhanov et al. (2004) in the Discussion (page 19, lines 4-6).

The authors don't explain that what they have found is peramorphosis - a condition where growth is continued to a later stage than occurred in the ancestor.

We thank the reviewer for this insightful comment. Given that the common ancestor of chicken and emu is presumed to have been volant with a keeled sternum, we interpret the loss of the keel and the early termination of TGF-β signaling in emu as an early truncation of the ancestral developmental program. Therefore, we believe the heterochronic shift in emu is better described as paedomorphosis rather than peramorphosis. We have clarified this point in the Discussion (page 19, lines 9-10).

> *The authors don't cite any reviews on heterochrony. They mention two experimental studies on the topic but many readers will not know much about heterochrony. So at least cite some reviews. E.g. the classic Gould 1977 Ontogeny and Phylogeny; or Syst Biol. 2005 Apr;54(2):230-40. doi: 10.1080/10635150590923227; or Semin Cell Dev Biol. 2014 Oct;34:99-107. doi: 10.1016/j.semcdb.2014.06.015.*

We thank the reviewer for this comment and we have now cited suggested publications in the Discussion (page 18, lines 20-21; page 19, line 9).

<u>Point-by-Point Response to the Reviewers</u> (2nd)

<u>Reviewer #1</u>

The authors have fully resolved my concerns and substantially improved the manuscript. The latest data well support the presented story and hypotheses. I appreciate the effort made by the authors during the revise period. This paper should be promptly published in Nature Communications.

We appreciate the reviewer's encouraging and careful assessment of our work.

<u>Reviewer #2</u>

My concerns have been addressed and I recommend publication.

We are grateful for the reviewer's assessment of our findings.

<u>Reviewer #3</u>

The authors have made a more than adequate rebuttal to the reviewers and so I now recommend publication without further revision. (Remarks on code availability):I haven't reviewed the code because this is outside my area of expertise.

We thank the reviewer for their thoughtful evaluation and recommendation.
